# A continuous ^13^C methacetin breath test for noninvasive assessment of intrahepatic inflammation and fibrosis in patients with chronic HCV infection and normal ALT

**DOI:** 10.1111/j.1365-2893.2008.01007.x

**Published:** 2008-10

**Authors:** G Lalazar, O Pappo, T Hershcovici, T Hadjaj, M Shubi, H Ohana, N Hemed, Y Ilan

**Affiliations:** 1 Liver Unit, Department of Medicine, Hadassah Hebrew University Medical CenterJerusalem, Israel; 2 Department of Pathology, Hadassah Hebrew University Medical CenterJerusalem, Israel

**Keywords:** breath test, liver fibrosis, methacetin, normal ALT

## Abstract

Up to 30% of patients with hepatitis C virus (HCV) infection and normal serum alanine aminotransferase (NALT) have significant liver disease. Currently, many of these patients undergo a liver biopsy to guide therapeutic decisions. The BreathID® continuous online ^13^C-methacetin breath test (MBT) reflects hepatic microsomal function and correlates with hepatic fibrosis. To assess its role in identifying intrahepatic inflammation and fibrosis in NALT patients, we tested 100 patients with untreated chronic HCV infection, and 100 age- and sex-matched healthy volunteers using ^13^C MBT following ingestion of 75 mg methacetin. All HCV patients had undergone a liver biopsy within 12 months of performing the MBT. Patients with a necroinflammatory grade ≤4 or >4, based on Ishak (modified HAI) score, HAIa + HAIb + HAIc + HAId, were defined as having low or high inflammation, respectively. Patients with a histological activity fibrosis stage ≤2 or >2, were defined as having nonsignificant or significant fibrosis, respectively. A proprietary algorithm to differentiate intrahepatic inflammation within chronic HCV patients with NALT achieved an area under the curve (AUC) of 0.90. Setting a threshold on the point of best agreement (at 83%) results in 82% sensitivity and 84% specificity. With application of another proprietary algorithm to differentiate patients with nonsignificant or significant fibrosis, 67% of liver biopsies performed in the patient group could have been avoided. This algorithm achieved an AUC of 0.92, with a sensitivity of 91% and a specificity of 88%. There was no correlation between body mass index (BMI) and MBT scores for patients with the same histological score. The continuous BreathID®^13^C MBT is an accurate tool for measuring the degree of inflammation and fibrosis in patients with chronic HCV infection and NALT. As such, it may prove to be a powerful, noninvasive alternative to liver biopsy in the management of this patient population.

## Introduction

The natural history of chronic hepatitis C virus (HCV) infection is variable, and some carriers have an indolent disease course with no complications even after decades of follow-up [1,2]. The decision to start treatment for chronic HCV infection depends on several factors, including: alanine aminotransferase (ALT) [3] levels, viral load, liver biopsy parameters of fibrosis and inflammation, patient determination and expected compliance [4]. The protracted course of the illness coupled with the intention of treating those who are most likely to benefit makes liver biopsy an important decision-making tool.

Serum ALT concentration, the most widely used indirect marker for liver disease activity, remains within the normal range in 25–30% of chronic HCV carriers, and an additional 40% have ALT levels less than twice the upper limit of normal [3,5]. It is generally accepted that the natural history of the subgroup of HCV carriers with persistently normal or minimally elevated ALT levels (NALT) is characterized by a slower progression rate [6–8]. Accordingly, follow-up and deferring therapy has been suggested in this patient group. A recent review of three large randomized trials has shown that patients with NALT have significantly lower inflammation and fibrosis scores on liver biopsy than patients with elevated ALT [9]. Nevertheless, these patients can have progressive liver disease and develop advanced fibrosis or cirrhosis [3,10]. These studies suggest that patients with chronic HCV with NALT should be evaluated similarly to patients with elevated ALT levels because they are at risk for developing significant liver disease [9].

Using periodic liver biopsies to determine if and when to use antiviral treatment is unlikely to elicit a favourable patient response and can lead to higher costs, increased cumulative cirrhosis incidence and decreased survival rates in comparison with ‘empirically based’ treatments [11,12]. Although considered the gold standard for assessment of liver fibrosis, liver biopsies have limitations, including inter-observer variability, sampling error and risks for complications. Reliable and inexpensive noninvasive methods to assess disease progression are a necessity in this setting [12].

Breath testing is based on the principal that an ingested substrate is metabolized, and a measurable metabolite is then expelled by the respiratory system. An ideal compound for this purpose is metabolized solely by the liver and therefore reflects liver function. Breath testing has been used experimentally and clinically for several decades [10], including for follow-up on patients with chronic liver disorders. The major drawbacks of these tests are the need for traditional, cumbersome isotopic ratio mass spectrometry methods, a prolonged testing time and patient inconvenience.

The BreathID® continuous online ^13^C-methacetin breath test (MBT), which reflects hepatic microsomal function (CYP1A2), is a laser-based technology that creates an infrared emission precisely matching the absorption spectrum of CO_2_ and can detect variations of less than 1/1000 in the ^13^CO_2_/^12^CO_2_ ratio measurement. The system is based on the measurement of CO_2_ by molecular correlation spectroscopy. This test offers several advantages: It is an office-based, noninvasive tool for the assessment of both liver inflammation and fibrosis does not involve a blood test and can provide an immediate result at the point-of-care. The aim of the present study was to determine its accuracy in assessing the degree of liver fibrosis and inflammation in patients with chronic HCV infection and NALT.

## Methods

### Study population

#### Patients

From 1 March 2006 to 31 May 2006, we enrolled 100 consecutive, unselected, patients with previously untreated, chronic HCV. All were anti-HCV and HCV RNA positive, with a normal serum ALT level (≤×2 ULN) on two separate tests during the preceding 6 months. All patients underwent a thorough physical examination and liver ultrasonography. Patients with ALT >×2 times the upper limit of the reference range were excluded. Patients were enrolled if they fulfilled the above criteria and had undergone a liver biopsy within 12 months of the breath test, as described below. Patients with other concomitant causes of liver disease such as hepatitis B virus (HBV), HIV, autoimmune hepatitis, alcohol abuse (excess of 40 g/day) and hepatocellular carcinoma were excluded from the study. Ultrasonographic evaluation of the abdomen was performed in all patients, and those with vessel occlusion were excluded.

#### Healthy volunteers

A group of 100 healthy volunteers (57 males and 43 females) were enrolled as controls in the study. They were screened by medical history, physical examination, liver ultrasound and routine liver function tests. All healthy volunteers had blood test results within normal limits. None had a history of active or previous liver disease or alcohol or drug abuse, and none were taking medications.

All participants gave written informed consent to their participation in the study, which was conducted in strict adherence to the principles of the Declaration of Helsinki. All experiments were approved by the Institutional Review Board committees and the Israel Ministry of Health Committee for Human Clinical Trials.

### Subject characteristics

Tables 1 & 2 show the main clinical, laboratory and histological characteristics of the patients and healthy volunteers at the time of liver biopsy, when applicable. The average age and body mass index (BMI) of the patients (36 females and 64 males) were 46 (SD 13.6; range 19–76) and 25.2 (SD 3.9; range 17.5–34.6), respectively. Difference in gender distribution between patients and healthy volunteers (chi-square test) was not significant. Comparing age and BMI between patients and healthy volunteers (*t*-tests) yields a significant difference (*P* = 0.0047) for age and a nonsignificant difference (*P* = 0.306) for BMI. For healthy volunteers, average age and BMI were 40.7 (SD 12.6, range 18–75) and 24.6 (SD 3.9, range 18–37), respectively. For HCV patients, average age and BMI were 46.3 (SD 13.6, range 19–76) and 25.3 (SD 4.0, range 17.5–34.6), respectively.

**Table 1:** Characteristics of patients and healthy volunteers

	Patients	Healthy volunteers
Number	100	100
Male	64	57
Female	34	43
Age	46.3 ± 13.6(19–76)	40.7 ± 12.6(18–75)*
BMI (kg/m^2^)	25.3 ± 3.95(17.5–34.6)	24.6 ± 3.9(18–37)
ALT (IU/mL)	57 ± 23	17.6 ± 8.1*
AST (IU/mL)	60 ± 26	21.3 ± 8.2*

* Values are presented as average ± SD (range). *P < 0.01.

**Table 2:** Patient clinical and laboratory parameters, divided by gender

	Gender		
	
Data	F	M	Overall
Count age	36	64	100
Average age	43.39	47.50	46.02
SD age	12.93	13.86	13.62
Min age	20.00	19.00	19.00
Max age	62.00	76.00	76.00
Average BMI	25.28	25.14	25.19
SD BMI	4.42	3.67	3.94
Min BMI	18.00	17.51	17.51
Max BMI	34.63	33.95	34.63
Average ALT	50.42	60.48	56.82
SD ALT	21.56	23.40	23.15
Min ALT	13.00	10.00	10.00
Max ALT	87.00	108.00	108.00
Average AST	61.42	58.78	59.74
SD AST	29.68	22.96	25.49
Min AST	19.00	22.00	19.00
Max AST	160.00	137.00	160.00
Average albumin	42.24	42.02	42.10
SD albumin	4.54	6.59	5.91
Min albumin	27.00	4.40	4.40
Max albumin	51.00	54.00	54.00
Average GGTP	48.40	65.07	58.80
SD GGTP	47.10	61.55	56.86
Min GGTP	9.00	13.00	9.00
Max GGTP	215.00	432.00	432.00
Average ALP	83.32	88.76	86.75
SD ALP	33.45	32.11	32.54
Min ALP	43.00	25.00	25.00
Max ALP	202.00	186.00	202.00
Average LDH	458.03	460.75	459.78
SD LDH	123.90	96.54	106.38
Min LDH	348.00	291.00	291.00
Max LDH	1073.00	929.00	1073.00
Average INR	1.02	1.11	1.08
SD INR	0.06	0.21	0.18
Min INR	1.00	1.00	1.00
Max INR	1.28	1.87	1.87
Average HGLB	14.06	15.60	15.04
SD HGLB	1.94	1.44	1.79
Min HGLB	8.60	9.60	8.60
Max HGLB	21.00	18.50	21.00
Average platelets	233.61	198.58	211.45
SD platelets	86.41	68.77	77.17
Min platelets	37.00	50.00	37.00
Max platelets	447.00	459.00	459.00
Average APRI	0.38	0.38	0.38
SD APRI	0.51	0.38	0.43
Min APRI	0.04	0.09	0.04
Max APRI	2.92	2.54	2.92
Average viral load	1 613 078.05	930 740.68	1 166 029.43
SD viral load	3 328 016.91	3 919 405.81	3 710 874.12
Average HAI fibrosis	2.53	3.02	2.84
SD HAI fibrosis	1.52	1.65	1.61
Min HAI fibrosis	0.00	0.00	0.00
Max HAI fibrosis	6.00	6.00	6.00

### Biochemical analysis

All patients underwent biochemical work-up, including a complete blood count, aspartate aminotransferase (AST), ALT(3), alkaline phosphatase, γ-glutamyltranspeptidase, lactate dehydrogenase, albumin, total bilirubin and prothrombin activity. Routine biochemical tests were performed using commercially available kits. The AST/ALT ratio and AST/platelet ratio index were calculated. For ALT measurements, an upper normal limit of 53 U/L was used (Table 2).

### Viral studies

All patients were found positive for anti-HCV by means of a third-generation ELISA (AxSYM HCV version 3.0; Abbott Laboratories, Abbott Park, IL, USA). Qualitative serum HCV-RNA detection was performed with reverse transcriptase-polymerase chain reaction in the 5′-noncoding region of the HCV genome (Roche COBAS Amplicor HCV Test, version 2.0: Roche Diagnostics, Basel, Switzerland). Quantification was performed using branched DNA with the Bayer’s VERSANT bDNA 3.0 assays (Bayer Diagnostics, Emeryville, CA, USA). The detection threshold was 3200 copies (615 IU) per mL. HCV genotyping was performed with INNO-LIPA HCV II (Innogenetics, Gent, Belgium).

### Liver histology

Following assessment of prothrombin time and platelet count, patients underwent a percutaneous, ultrasound-guided liver biopsy under local anaesthesia (lignocaine 1%). Specimens obtained by means of Menghini needles, diameter 1.6 mm, had an average length of 20 ± 5 mm (range, 15–25 mm), and representative according to accepted standards. Biopsy specimens were fixed with formalin, embedded in paraffin and stained with haematoxylin and eosin. All sections were reviewed by an expert pathologist blinded to patient clinical data and breath-test results.

Necroinflammatory score was graded using the HAI score based on periportal or periseptal interface hepatitis (piecemeal necrosis) (0–4), confluent necrosis (0–6), focal (spotty) lytic necrosis, apoptosis, and focal inflammation (0–4) and portal inflammation (0–4) [13]. Fibrosis was staged using the Ishak (modified HAI) fibrosis score on a scale from 0 to 6 [13]. Table 3 shows selected patient data grouped by fibrosis score.

**Table 3:** HCV patient population grouped by modified HAI fibrosis stage for patient data and blood test results

	Modified HAI fibrosis stage							
		
Data	0	1	2	3	4	5	6	Total
Count	4	14	32	24	6	10	10	100
Average age	32.25	42.36	41.97	49.21	50.50	53.10	52.20	46.02
SD age	7.37	14.06	13.91	13.27	13.22	10.97	9.98	13.62
Min age	27.00	19.00	20.00	23.00	34.00	35.00	35.00	19.00
Max age	43.00	64.00	70.00	76.00	68.00	74.00	68.00	76.00
Average BMI	21.59	24.61	24.14	26.49	24.26	26.22	27.23	25.19
SD BMI	2.19	4.28	3.61	3.42	3.19	3.54	5.35	3.94
Min BMI	19.13	19.14	17.51	21.04	19.75	20.82	20.06	17.51
Max BMI	24.44	32.41	32.87	34.63	28.37	31.12	33.95	34.63
Average ALT	39.25	41.36	57.87	55.25	62.33	70.20	70.30	56.92
SD ALT	34.65	22.05	20.71	22.25	14.95	27.37	18.60	23.39
Average AST	35.75	45.00	51.52	61.00	67.17	74.20	93.50	59.74
SD AST	11.38	11.38	14.83	28.99	25.54	23.80	25.86	25.49
Average albumin	41.75	44.42	42.96	41.76	43.17	42.56	36.80	42.10
SD albumin	3.86	3.06	3.78	8.98	5.49	3.50	5.20	5.91

### Noninvasive breath testing

Following an overnight (>8 h) fast, patients and healthy volunteers were connected to the breath-testing unit’s BreathID® system (BreathID Ltd, Jerusalem, Israel) via nasal cannula (IDcircuit^TM^), and received 75 mg of *N*-(4-methoxy-^13^C-phenyl)acetamide (methacetin, Isotec) dissolved in 150 mL of water. Breath samples were collected using an automatic breath sampling unit under continuous capnographic control, before and for 60 min after the labelled substrate was administered to the patient. The ^13^CO_2_/^12^CO_2_ ratios in the breath samples were determined and mapped on the screen at a high frequency (once every 2–3 min). During the test period, all patients and healthy volunteers continued fasting and were at rest to eliminate any variability in CO_2_ excretion due to the ingestion of food or physical activity.

### Analysis of breath-test data

Results obtained from the device were expressed as percentage of administered dose of ^13^C per cent dose recovered (PDR) and the cumulative PDR (CPDR) percentage of ^13^C recovered over time at 10, 15, 20, 30 and 60 min after ingestion of methacetin, respectively, as well as the PDR peak and peak time. PDR refers to the rate at which the ^13^C substrate is metabolized and is expressed in %/h. PDR expresses the rate of substrate metabolization derived from the change in the ^13^C/^12^C ratio, in which the specific test details are taken into account, thereby normalizing the results and making them independent of differences in weight, height, dose or substrate type and purity [14,15]. CPDR is the numeric integral of PDR and describes the total amount of substrate metabolized at any given accumulated time. Data are expressed in units of %/h for PDR and per cent for CPDR. The BreathID® device plots the PDR and CPDR in real-time and provides PDR peak value and peak time.

### Statistical analysis

Using Spearman’s nonparametric Rho correlation, the correlation between the different breath-test parameters and modified Ishak HAI inflammation and fibrosis scores, gender, BMI and age were assessed. Patients were grouped according to fibrosis scores, using breath-test parameters to compare between HAI fibrosis scores of ≤2 *vs* >2, and HAI inflammation scores (HAIa + HAIb + HAIc + HAId) ≤4 and >4, respectively. Mann–Whitney’s two-samples test and logistic regression with receiver operating characteristic (ROC) curve analysis were used to evaluate the ability of different breath-test parameters and their combination to predict the severity of fibrosis and inflammation. Finally, the repeatability of the test was determined by assessing several participants more than once during a period of <2 weeks.

Two algorithms which include several breath-test parameters and patient data were developed to allow differentiation of high *vs* low inflammation, and significant *vs* nonsignificant fibrosis, with high sensitivities and specificities while maximizing the number of liver biopsies identified as avoidable.

## Results

### Breath-test parameters significantly differentiate grade of intrahepatic necroinflammation in chronic HCV patients with NALT

The Mann–Whitney ‘two-samples test’, used to compare inflammation groups (HAIa + HAIb + HAIc + HAId ≤ 4 *vs* > 4) for each breath-test parameter, yielded significant (*P* < 0.005) results for selected breath-test parameters (Table 4). A binary logistic regression analysis was performed with high/low inflammation as the dependent variable and breath-test parameters as explanatory variables, controlled by age, BMI and gender.

**Table 4:** Comparing between BT parameters and degree of intrahepatic inflammation for HAIa + HAIb + HAIc + HAId ≤ 4 *vs* > 4

Breath-test parameters		*n*	Mean	SD	SE	Asymp. sig. (two-tailed) Mann–Whitney test
PDR peak	Low inflammation	32	38.295	15.7333	2.7813	0.0063
	High inflammation	68	28.589	11.1991	1.3581	
Peak time	Low inflammation	32	18.861	7.6213	1.3473	0.0477
	High inflammation	68	22.831	9.4697	1.1484	
PDR10	Low inflammation	32	27.274	16.5672	2.9287	0.0148
	High inflammation	68	18.982	11.5059	1.3953	
PDR15	Low inflammation	32	33.894	14.8954	2.6332	0.0034
	High inflammation	68	23.764	12.1687	1.4757	
PDR20	Low inflammation	32	32.586	11.2144	1.9824	0.0034
	High inflammation	68	24.602	10.6473	1.2912	
PDR30	Low inflammation	32	26.339	7.6357	1.3498	0.0170
	High inflammation	68	21.714	7.7831	0.9438	
PDR60	Low inflammation	32	14.749	3.8186	0.6750	0.1105
	High inflammation	68	12.906	3.9964	0.4846	
CPDR10	Low inflammation	32	2.348	1.5852	0.2802	0.0072
	High inflammation	68	1.510	0.9802	0.1189	
CPDR15	Low inflammation	32	4.825	2.7483	0.4858	0.0076
	High inflammation	68	3.255	1.9102	0.2316	
CPDR20	Low inflammation	32	7.500	3.6267	0.6411	0.0049
	High inflammation	68	5.204	2.7542	0.3340	
CPDR30	Low inflammation	32	12.502	4.7985	0.8483	0.0028
	High inflammation	68	9.136	4.0145	0.4868	
CPDR60	Low inflammation	32	22.255	6.4667	1.1432	0.0042
	High inflammation	68	17.564	6.0341	0.7317	

### Breath-test parameters significantly differentiate degree of fibrosis on liver histology in chronic HCV patients with NALT

Most of breath-test parameters evaluated showed a statistically significant (*P* < 0.005) difference between the two modified Ishak HAI fibrosis stages. Because therapeutic decisions are based on the histological level of fibrosis, the ability of the MBT to stage fibrosis was assessed. The Mann–Whitney ‘two-sample tests’ was used to compare the level of significance for each breath-test parameter and the modified Ishak HAI fibrosis stage. Patients were grouped into nonsignificant fibrosis (modified Ishak HAI stages ≤ 2, *n* = 50) and significant fibrosis (modified Ishak HAI stages > 2, *n* = 50) categories. MBT parameters were found to be statistically significant in differentiating both fibrosis groups. Data are summarized in Table 5. To develop a diagnostic mathematical model, logistic regression was used with significant/nonsignificant fibrosis assessed with the modified Ishak HAI fibrosis stages as the dependent variable and breath-test variables as explanatory variables, controlled by age, BMI and gender.

**Table 5:** MBT data grouped by fibrosis groupings (nonsignificant HAIf ≤ 2/significant HAIf > 2) including significance

Breath-test parameter	Fibrosis	*n*	Mean	SD	SE	Asymp. Sig. (two-tailed) Mann–Whitney test
PDR peak	Nonsignificant	50	36.843	11.3709	1.6081	<0.0001
	Significant	50	26.547	13.6582	1.9316	
Peak time	Nonsignificant	50	19.860	7.2024	1.0186	0.2022
	Significant	50	23.261	10.4207	1.4737	
PDR10	Nonsignificant	50	25.135	12.6433	1.7880	0.0030
	Significant	50	18.136	14.1545	2.0017	
PDR15	Nonsignificant	50	31.563	12.3783	1.7506	0.0009
	Significant	50	22.448	13.8851	1.9636	
PDR20	Nonsignificant	50	31.875	9.8685	1.3956	<0.0001
	Significant	50	22.438	10.9569	1.5495	
PDR30	Nonsignificant	50	26.842	6.3321	0.8955	<0.0001
	Significant	50	19.546	7.8717	1.1132	
PDR60	Nonsignificant	50	15.131	3.2787	0.4637	0.0002
	Significant	50	11.861	4.0458	0.5722	
CPDR10	Nonsignificant	50	2.101	1.2012	0.1699	0.0017
	Significant	50	1.455	1.2489	0.1766	
CPDR15	Nonsignificant	50	4.404	2.0923	0.2959	0.0017
	Significant	50	3.110	2.3730	0.3356	
CPDR20	Nonsignificant	50	6.963	2.8366	0.4012	0.0006
	Significant	50	4.915	3.2934	0.4658	
CPDR30	Nonsignificant	50	11.932	3.7767	0.5341	<0.0001
	Significant	50	8.495	4.6195	0.6533	
CPDR60	Nonsignificant	50	22.015	4.8239	0.6822	<0.0001
	Significant	50	16.115	6.7072	0.9485	

### Breath-test parameters significantly differentiate between chronic HCV patients with NALT and healthy volunteer groups

**Fig. 1:**
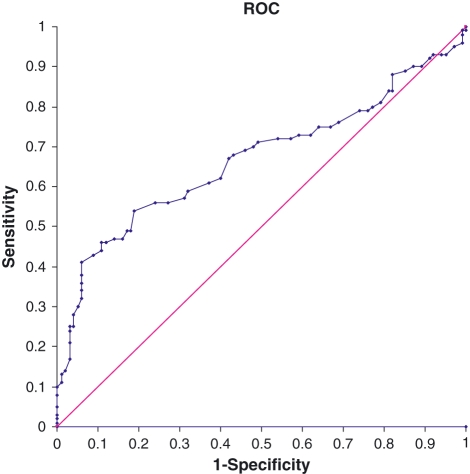
A binary logistic regression model using BreathID and demographic parameters (*P* < 0.001 for each of the parameters) showed that the methacetin breath test (MBT) can differentiate patients and healthy volunteers with an area under the curve (AUC) of 0.7 (95% CI, 0.626–0.778), sensitivity of 56% and specificity of 86%. A binary logistic regression model using BreathID® and demographic parameters (*P* < 0.001 for each of the parameters) showed that the MBT can differentiate patients and healthy volunteers with an AUC of 0.67 (95% CI, 0.59–0.74), sensitivity of 56% and specificity of 86%. Using *Charles E. Metz* ROCKIT 1.1B2 provides the following results: binormal parameters and area under the estimated ROC: a, 0.5158; b, 0.5824; area (Az), 0.6721; area (Wilc), 0.6689. Estimated standard errors (SE) and correlation of these values: SE (a), 0.1256; SE (b), 0.0754; corr (a,b), 0.2129; SE (Az), 0.0384; SE (Wilc), 0.0380; symmetric 95% CI for a, (0.2697, 0.7619); b, (0.4347, 0.7301); asymmetric 95% CI for Az, (0.5938, 0.7435).

A binary logistic regression model using PDR20 and age (*P* < 0.001 for each of the two parameters) showed that the MBT can differentiate patients and healthy volunteers with an area under the curve (AUC) of 0.67 (95% CI, 0.59–0.74), sensitivity of 56% and specificity of 86% (Table 6, Fig. 1).

**Table 6:** Values of all MBT parameters differed significantly between healthy volunteers and subsets of subjects with HCV infection

MBT parameter	Healthy volunteer	HCV total	Modified HAIf ≤ 2	Modified HAIf ≥ 2
PDR peak	35.31 ± 8.94	31.7 ± 13.53	36.84 ± 11.37	26.55 ± 13.66
Peak time	21.04 ± 7.47	21.56 ± 9.07	19.86 ± 7.2	23.26 ± 10.42
PDR10	23.85 ± 10.78	21.64 ± 13.81	25.14 ± 12.64	18.14 ± 14.15
PDR15	30.63 ± 10.67	27.01 ± 13.87	31.56 ± 12.38	22.45 ± 13.89
PDR20	32.18 ± 8.65	27.16 ± 11.41	31.88 ± 9.87	22.44 ± 10.96
PDR30	27.03 ± 5.29	23.19 ± 8	26.84 ± 6.33	19.55± 7.87
PDR60	15.82 ± 2.6	13.5 ± 4.02	15.13 ± 3.28	11.86 ± 4.05
CPDR10	1.92 ± 0.92	1.78 ± 1.26	2.1 ± 1.2	1.46 ± 1.25
CPDR15	4.12 ± 1.75	3.76 ± 2.32	4.4 ± 2.09	3.11 ± 2.37
CPDR20	6.66 ± 2.44	5.94 ± 3.23	6.96 ± 2.84	4.91 ± 3.29
CPDR30	11.7 ± 3.3	10.21 ± 4.54	11.93 ± 3.78	8.49 ± 4.62
CPDR60	21.9 ± 4.11	19.07 ± 6.52	22.02 ± 4.82	16.12 ± 6.71

### Assessment of serum blood test parameters

Laboratory parameters such as ALT, albumin, prothrombin time, international normalized ratio levels and the AST/platelet ratio index score were analysed for dependence with the modified Ishak HAI fibrosis groups. Results are summarized in Table 7. Since most models using blood-test parameters to assess liver disease are often based on elevated ALT values, they failed in the patient population studied in the present study.

**Table 7:** Comparison of laboratory results based on fibrosis grouping: significance of laboratory tests in differentiating between high and low fibrosis groups

Breath-test parameter	Fibrosis	*n*	Mean	SD	SE	Asymp. Sig. (two-tailed) Mann–Whitney test
ALT	Nonsignificant	49	51.633	23.3146	3.3307	0.0453
	Significant	50	62.100	22.5065	3.1829	
AST	Nonsignificant	49	48.367	14.2693	2.0385	<0.0001
	Significant	50	70.880	29.0542	4.1089	
Albumin	Nonsignificant	44	43.250	3.6096	0.5442	0.0921
	Significant	48	41.050	7.3026	1.0540	
GGTP	Nonsignificant	46	41.630	29.7137	4.3810	0.0010
	Significant	47	75.596	70.8485	10.3343	
ALP	Nonsignificant	44	72.180	18.8024	2.8346	0.0001
	Significant	48	100.104	36.6787	5.2941	
LDH	Nonsignificant	41	442.341	60.5077	9.4497	0.3474
	Significant	46	475.326	133.6057	19.6991	
INR	Nonsignificant	46	1.040	0.1434	0.0211	0.0017
	Significant	49	1.109	0.1977	0.0282	
HGLB	Nonsignificant	48	15.031	1.4531	0.2097	0.6087
	Significant	50	15.040	2.0829	0.2946	
APRI	Nonsignificant	48	0.215	0.0912	0.0132	<0.0001
	Significant	50	0.539	0.5554	0.0785	
Platelets	Nonsignificant	48	239.167	59.8023	8.6317	<0.0001
	Significant	50	184.840	82.9741	11.7343	

### Correlation with patient characteristics

No significant correlation was shown between gender and modified Ishak HAI fibrosis stages (chi-square analysis, *P* = 0.145). When compared by two-tailed *t*-tests, BMI and age differed significantly (*P* = 0.004 and *P* < 0.001, respectively) between significant and nonsignificant fibrosis (HAIf < 3 or HAIf ≥ 3). Spearman’s Rho correlations showed that some breath-test parameters were correlated with age (PDR peak, PDR20, CDPR30, CPDR60) or BMI (PDR peak, CPDR60).

### Repeatability of breath testing

A total of 42 healthy volunteers and 11 patients were assessed for test repeatability using the ‘within-subject coefficient of variation’ of MBT parameters (Table 8). The number of repetitions per person was between two and six. Estimations were based on four BT parameters for healthy volunteers/patients separately and for both groups together. Repeating the breath testing in both patients and in healthy volunteers (repeats ≥2) resulted in an inter-test variability of ≤13% for the PDR peak height (95% CI, 0.11–0.15).

**Table 8:** Calculated reproducibility values for selected groups

	CV	Lower 95% bound	Higher 95% bound
Healthy subjects (*n* = 42)			
PDR peak	0.1259	0.1035	0.1483
Peak divided by time	0.3619	0.3024	0.4213
PDR20	0.1644	0.1355	0.1932
CPDR20	0.2392	0.1983	0.2801
Patients (*n* = 11)			
PDR peak	0.1501	0.1004	0.1997
Peak divided by time	0.3043	0.2079	0.4007
PDR20	0.1771	0.1190	0.2352
CPDR20	0.2362	0.1600	0.3125
All subjects (*n* = 53)			
PDR Peak	0.1317	0.1111	0.1522
Peak divided by time	0.3494	0.2989	0.3999
PDR20	0.1673	0.1415	0.1931
CPDR20	0.2385	0.2027	0.2743

### The use of MBT as a tool to avoid the need for liver biopsy

#### Necroinflammation

**Fig. 2:**
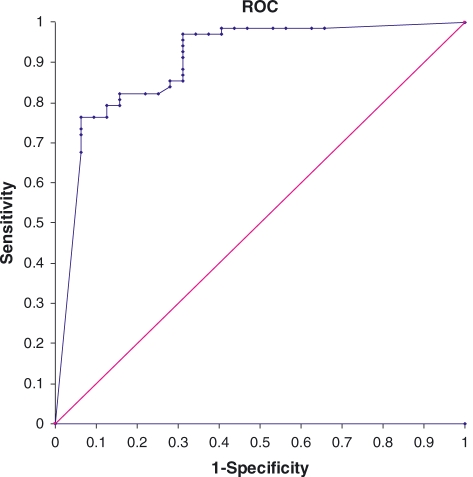
Model to differentiate between HAIa + HAIb + HAIc + HAId ≤ 4 *vs* HAIA + HAIB + HAIC + HAID > 4: A proprietary algorithm that includes breath-test parameters, age and other patient data to differentiate intrahepatic inflammation (HAIa + HAIb + HAIc + HAId ≤ 4 *vs* > 4) within chronic hepatitis C virus (HCV) patients with normal alanine aminotransferase (NALT) achieved an area under the curve (AUC) of 0.90. Setting a threshold at the point of best agreement (at 83%), results in 82% sensitivity and 84% specificity. At the dataset’s prevalence of 68% the PPV is 92% and the NPV is 69%. Assuming a prevalence of 45.5% would lead to a PPV of 82% and an NPV of 85%. Using *Charles E. Metz* ROCKIT 1.1B2 provides the following results: binormal parameters and area under the estimated ROC: a, 1.9574; b, 1.0126; area (Az), 0.9155; area (Wilc), 0.9021. Estimated standard errors (SE) and correlation of these values: SE (a), 0.3453; SE (b), 0.2961; corr (a,b), 0.6238; SE (Az), 0.0305; SE (Wilc), 0.0297. Symmetric 95% confidence intervals: For a, (1.2807, 2.6342); for b, (0.4322, 1.5930); asymmetric 95% CI for Az, (0.8389, 0.9609).

By using a proprietary algorithm that includes breath-test parameters, age and other patient data to differentiate intrahepatic inflammation (HAIa + HAIb + HAIc + HAId ≤ 4 *vs* > 4) for chronic HCV patients with NALT, an AUC of 0.90 was achieved (Fig. 2). Setting a threshold on the point of best agreement (at 83%) results in sensitivity of 82% and specificity of 84%. At the dataset’s prevalence of 68%, the positive predictive value (PPV) was 92% and the negative predictive value (NPV) was 69%. Assuming a prevalence of 45.5%, this would lead to a PPV of 82% and an NPV of 85%.

#### Fibrosis

**Fig. 3:**
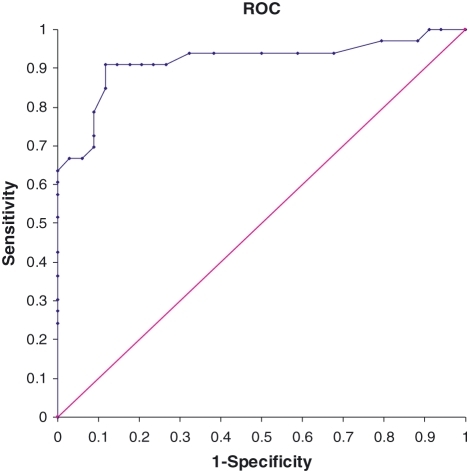
Receiver operating characteristic (ROC) curve describing performance of the 67% patients where significant/nonsignificant fibrosis was determined: Using *Charles E. Metz* ROCKIT 1.1B2 provides the following results: binormal parameters and area under the estimated ROC: a, 1.4888; b, 0.4950; area (Az), 0.9090; area (Wilc), 0.9153; estimated standard errors (SE) and correlation of these values: SE (a), 0.3047; SE (b), 0.1617; corr (a,b), 0.5744; SE (Az), 0.0384; SE (Wilc), 0.0365; symmetric 95% CI: For a, (0.8916, 2.0861); for b, (0.1782, 0.8118); asymmetric 95% CI for Az, (0.8091, 0.9636).

By using an algorithm that includes breath-test parameters, age and other patient data, 67% of liver biopsies performed in the patient group could have been avoided (Fig. 3). This algorithm achieved an AUC of 0.92, with a sensitivity of 91% and a specificity of 88%, a PPV of 88% and an NPV of 91%. Thirty-four patients were identified as having significant fibrosis, including four false positives: two with a HAI fibrosis score of 2, and an additional two with a score of 1. Thirty-three patients were identified as having nonsignificant fibrosis, including three false negatives: two with a HAI fibrosis score of 3 and one with a score of 5. There was no correlation between age or BMI and MBT scores for patients with the same histological score.

**Fig. 4:**
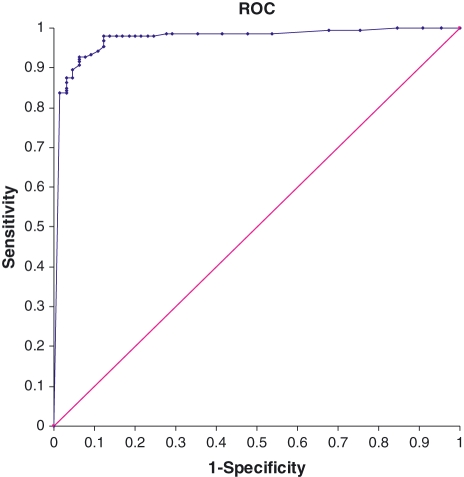
By using the same proprietary algorithm developed to differentiate significant from nonsignificant fibrosis, 65% of the tested subjects would get an answer. Using *Charles E. Metz* ROCKIT 1.1B2 provides the following results: binormal parameters and area under the estimated ROC: a, 1.8231; b, 0.9943; area (Az), 0.9020; area (Wilc), 0.9005; estimated standard errors (SE) and correlation of these values: SE (a), 0.3697; SE (b), 0.2406; corr(a,b), 0.7157; SE (Az), 0.0322; SE (Wilc), 0.0378; symmetric 95% CI for a, (1.0984, 2.5477); for b, (0.5226, 1.4660); asymmetric 95% CI for Az, (0.8234, 0.9513).

Applying the same proprietary algorithm developed to differentiate significant from nonsignificant fibrosis on the healthy volunteer group combined with the significant fibrosis group (*n* = 150), 67% of the tested subjects (*n* = 98) would get an answer (Fig. 4). This algorithm achieved an AUC of 0.92, with a sensitivity of 91% and a specificity of 88%, a PPV of 79% and NPV of 95%. Thirty-eight subjects were identified as having significant fibrosis, including eight false positives. Sixty subjects were identified as having nonsignificant fibrosis including three false negatives; two with a HAI fibrosis score of 3 and one with a score of 5.

#### Combination of inflammation and fibrosis algorithms

**Fig. 5:**
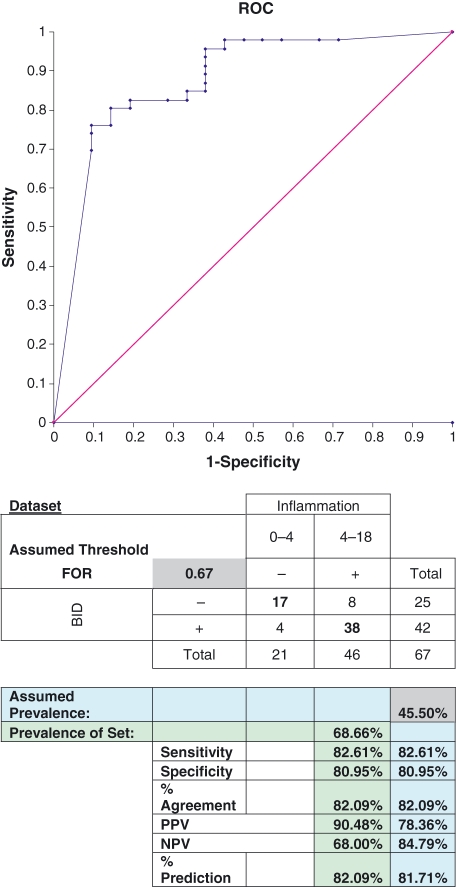
A proprietary algorithm that includes breath-test parameters, age and other patient data to differentiate intrahepatic inflammation (HAIa + HAIb + HAIc + HAId ≤ 4 *vs* > 4) applied on the 67% of the patient population assessed by the fibrosis algorithm, yields an area under the curve (AUC) of 0.89. Leaving the threshold at the point of best agreement (at 83%) found in the inflammation algorithm, results in 83% sensitivity and 81% specificity. At the dataset’s prevalence of 68%, the PPV is 91% and the NPV is 68%. Assuming a prevalence of 45.5%, this leads to a PPV of 78% and an NPV of 85%. Using *Charles E. Metz* ROCKIT 1.1B2 provides the following results: binormal parameters and area under the estimated ROC: a, 1.6781; b, 0.8979; area (Az), 0.8941; area (Wilc), 0.8737; estimated standard errors (SE) and correlation of these values: SE (a), 0.3529; SE (b), 0.3125; corr(a,b), 0.5320; SE (Az), 0.0419; SE (Wilc), 0.0419; symmetric 95% CI: for a, (0.9865, 2.3698); for b, (0.2855, 1.5103); asymmetric 95% CI for Az, (0.7882, 0.9552).

Applying the described inflammation algorithm on the subset of patients analysed by the fibrosis algorithm (67% of the initial population), resulted in an area under the ROC of 0.89. When the same threshold was used, sensitivity and specificity were 83 and 81% respectively, with PPV and NPV of 91 and 68%, respectively (Fig. 5).

**Fig. 6:**
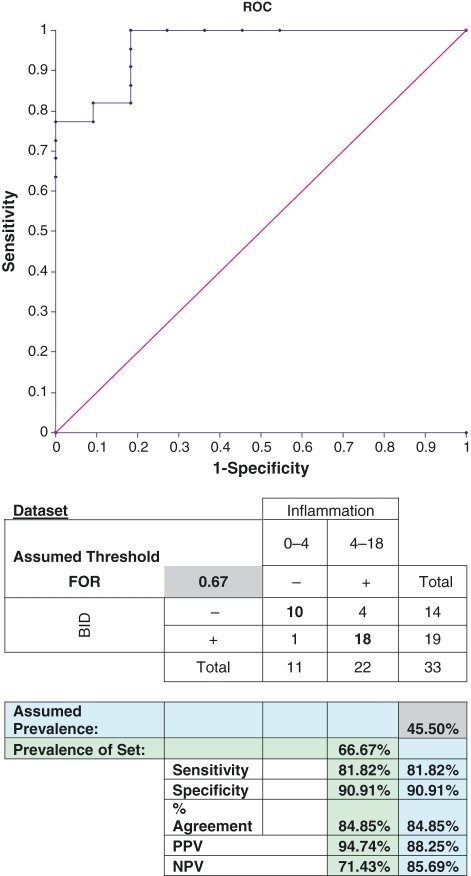
A proprietary algorithm that includes breath-test parameters, age and other patient data to differentiate intrahepatic inflammation (HAIa + HAIb + HAIc + HAId ≤ 4 *vs* > 4) applied on the 33% of patient population not assessed by the fibrosis algorithm yields an area under the curve (AUC) of 0.96. Leaving the threshold on the point of best agreement (at 83%) found in the inflammation algorithm results in 82% sensitivity and 91% specificity. At the dataset’s prevalence of 68% the PPV is 95% and the NPV is 71%. Assuming a prevalence of 45.5%, this leads to a PPV of 88% and an NPV of 86%. Using *Charles E. Metz* ROCKIT 1.1B2 provides the following results: binormal parameters and area under the estimated ROC: a, 3.5668; b, 1.7357; area (Az), 0.9625; area (Wilc), 0.9628; estimated standard errors (SE) and correlation of these values: SE (a), 1.9626; SE (b), 1.5130; corr (a,b), 0.9003; SE (Az), 0.0419; SE (Wilc), 0.0314. Symmetric 95% CI: For a, (−0.2800, 7.4135); for b, (−1.2298, 4.7013); asymmetric 95% CI for Az, (0.7814, 0.9973).

Applying the described inflammation algorithm on the subset of patients not analysed by the fibrosis algorithm (33% of the initial population), resulted in an area under the ROC of 0.96. When the same threshold was used, sensitivity and specificity were 82 and 91%, respectively, with PPV and NPV of 95 and 71%, respectively (Fig. 6).

## Discussion

We assessed the ability of the noninvasive, online, continuous ^13^C-MBT in the detection of significant fibrosis and inflammation in patients with HCV. In a cohort of 100 consecutive HCV patients with normal ALT, breath-test parameters correlated with the level of fibrosis and degree of inflammation as indicated by the modified Ishak HAI fibrosis and inflammation scores. The breath test accurately differentiated low and high inflammation (≤4 and >4, 83%). The MBT achieved a 90% diagnostic accuracy in differentiating patients with a modified Ishak HAI fibrosis score ≤2 and >2 while 67% of the biopsies could have been avoided by replacing the assessment with the MBT alone.

Methacetin breath testing has been correlated with fibrosis and overall liver function [16]. Traditionally, this testing was performed using isotopic ratio mass spectrometry, the gold standard for MBT. However, a recent study found that a measurement method with continuous automatic molecular correlation spectroscopy showed a high correlation with mass spectroscopy [17]. In addition to being less cumbersome, the continuous system has an inherent advantage over mass spectrometry in its ability to identify the PDR peak and PDR peak time, which are often missed when noncontinuous measurement is used. Furthermore, being fully automatic and using an internal capnograph, the system mitigates the risk of potential human errors and ensures that the appropriate part of the breath sample is collected.

Several noninvasive methods have been explored as tools to assess the degree of liver fibrosis in chronic HCV patients, and some were also evaluated in patients with normal ALT. These include a combination of serum tests such as the AST/ALT ratio [18] or the AST/platelet ratio index [19]. To date, two methods appear to be the most studied with the goal of using them to supersede liver biopsy in the assessment of liver fibrosis. The first is a patented artificial intelligence algorithm (Fibrotest®; BioPredictive, Paris, France) [20,21]. The second is a technique to measure *in vivo* liver elasticity, based on one-dimensional transient elastography (Fibroscan®, EchoSens, Paris, France) [22,23].

The Fibrotest® requires a blood sample and specialized laboratory, which in turn translates into a lag time of several days between test and result. Estimation of liver fibrosis by Fibrotest® uses five parameters that were chosen by logistic regression applied to a selection of basic serum biochemical markers, with histological staging as the independent variable [20]. Mean ALT values were three times the upper limit of the reference range for males, and only 13% of the studied patients had ALT within the normal range. Biochemical markers were measured once on the day of biopsy, but because ALT fluctuates widely during the course of chronic HCV infection, it is likely that only a few, if any, were HCV carriers with NALT. In a subsequent Fibrotest® prospective validation study, participants needed documented elevated serum ALT levels (at least 1.5 times the upper limit of normal) on three occasions within 6 months before enrolment. There have been very few independent studies using Fibrotest®. In addition to inter-laboratory variations, these studies have shown that in about 15–20% of patients, significant fibrosis could be missed or conversely, significant fibrosis could be diagnosed in the presence of minimal or no fibrosis [24]. In patients with Gilbert syndrome, or any acute inflammation with high haptoglobin values, higher false-positive and false-negative rates were found. In a recent study of 40 patients with NALT, Fibrotest® had an accuracy of only 43%, with a sensitivity and specificity of only 64 and 31%, respectively (11). Both Fibrotest® (measuring fibrosis stage in patients with chronic HCV or HBV) and ActiTest® (measuring necroinflammatory activity in patients with chronic HCV or HBV) are dependent on inter-laboratory variability of biochemical markers [25].

The Fibroscan® provides a noninvasive method for assessing liver fibrosis but does not give information regarding inflammation. In addition, it can be difficult to administer and may produce imprecise results in obese patients. Measurement of liver elasticity so far has been precluded by technical limitations and costs. With Fibroscan®, a transmitted elastic wave can be temporally separated from reflected elastic waves, making the technique less sensitive to those boundary conditions (including body fat) that tend to induce artefacts [22].

All the currently used noninvasive methods have a diagnostic accuracy that does not exceed 80–85% [26–29]. Thus, many patients still require a liver biopsy, and in those classified without one, misdiagnosis is expected in at least 15–20% [30]. The ability of the MBT to accurately assess the degree of intrahepatic inflammation and fibrosis in patients with NALT may add to its value in decision making for these patients.

The clinical management of chronic HCV infection is based on both patient and viral characteristics, and a liver biopsy is often required to guide therapeutic decision making. Paradoxically, patients with NALT, in whom liver biopsy is particularly useful, are more reluctant to undergo one. An attempt to increase the diagnostic performance of noninvasive markers of liver fibrosis by combining them in sequential algorithms was recently suggested. Recently 190 patients with chronic HCV were evaluated for AST-to-platelets ratio, Forns’ index and Fibrotest® results at the time of liver biopsy, and stepwise combination algorithms were developed and validated prospectively in 100 additional patients. The data suggested that a stepwise combination of noninvasive markers of liver fibrosis improves diagnostic performance in chronic HCV, reducing the need for a liver biopsy [31]. The data of the present study show that by using an algorithm that includes breath-test parameters, age and other patient data, 67% of liver biopsies performed in the patient group could have been avoided. This algorithm achieved an AUC of 0.92, with a sensitivity of 91% and a specificity of 88%.

As novel therapies for liver fibrosis evolve, noninvasive measurement of liver fibrosis will be required to help manage patients with chronic liver disease. The BreathID® holds several advantages as a noninvasive tool in this setting, including not being limited by patient BMI or other patient characteristics, such as the presence of Gilbert syndrome or acute inflammatory condition. The test provides information on both fibrosis and inflammation. Future studies will determine its correlation with the functional hepatic mass and hepatic reserve along with the clinical course in these patients.

The results of the current study suggest that the continuous BreathID®^13^C MBT is an accurate tool for identification of liver inflammation and fibrosis in patients with chronic HCV infection and normal ALT levels, and that its use can avoid the need for a liver biopsy in two-thirds of these patients. As such, it may prove to be a powerful, noninvasive alternative for decision making in the management of this patient population.

## Abbreviations:

ALT: alanine aminotransferase
AUC: area under the curve
BMI: body mass index
CPDR: cumulative per cent dose recovered
HCV: hepatitis C virus
MBT: methacetin breath test
NALT: normal alanine aminotransferase
PDR: per cent dose recovered
ROC: receiver operating characteristic
SD: standard deviation
